# Investigating the Risk Factors Associated with Type 1 Diabetes (T1D) in the South of Iran: A Case-control Study

**DOI:** 10.2174/0115733998336000241120104439

**Published:** 2025-01-03

**Authors:** Fatemeh Sadat Mirrashidi, Amin Kiani, Rasoul Raesi, Kiavash Hushmandi, Salman Daneshi

**Affiliations:** 1 Department of Pediatrics, School of Medicine Imam Khomeini Hospital Jiroft University of Medical Sciences, Jiroft, Iran;; 2 Department of Nursing, School of Nursing and Midwifery Jiroft University of Medical Sciences, Jiroft, Iran;; 3 Department of Nursing, Torbat Jam Faculty of Medical Sciences, Torbat Jam, Iran;; 4 Department of Health Services Management, School of Health, Mashhad University of Medical Sciences, Mashhad, Iran;; 5 Nephrology and Urology Research Center, Clinical Sciences Institute, Baqiyatallah University of Medical Sciences, Tehran, Iran;; 6 Department of Public Health, School of Health, Jiroft University of Medical Sciences, Jiroft, Iran

**Keywords:** Type 1 diabetes, gene environment interaction, environmental exposure, hyperglycemia, protective factors, etiology, primary prevention, diabetes

## Abstract

**Introduction:**

Type 1 diabetes (T1D) is an autoimmune disorder characterized by a complex interplay of genetic and environmental factors.

**Aim:**

The objective of this study was to identify the risk factors associated with T1D in the southern region of Iran during the year 2022.

**Methods:**

This research employed a case-control design involving two groups (79 individuals in each group) of healthy children and adolescents diagnosed with T1D. The study assessed and compared the groups regarding various potential risk factors that may influence the development of T1D. Data analysis was performed using SPSS-22 software.

**Results and Discussion:**

Significant differences were observed between the two groups concerning several factors, including the age at which children began kindergarten, their weight at one year and 18 months, maternal weight gain during pregnancy, delivery method, age of introduction to complementary feeding, duration of breastfeeding, use of cow's milk and vitamin D supplements before one year of age, as well as family history of T1D and other autoimmune diseases among fathers and siblings.

**Conclusion:**

The findings indicate that, in addition to genetic predispositions, numerous environmental factors contribute to the risk of developing T1D. Consequently, it is recommended that health managers and policymakers investigate these risk factors more broadly across various regions to implement effective strategies aimed at reducing the incidence of T1D nationwide.

## 1 INTRODUCTION

Type 1 diabetes (T1D) is a chronic autoimmune disease that occurs due to the destruction of pancreatic beta cells, resulting in a reduction or absence of insulin. Consequently, patients require external insulin for the rest of their lives. In this type of diabetes, unlike in type 2 diabetes (T2D), insulin resistance (IR) is typically not observed [1, 2]. Recent research has increasingly highlighted the role of gut microbiotas as a significant contributor to insulin resistance (IR). Studies indicate that alterations in gut microbial composition, particularly increased carbohydrate metabolism by specific bacterial species, are linked to elevated levels of host-accessible monosaccharides [3, 4]. These monosaccharides can promote lipid accumulation and activate inflammatory responses, which further exacerbate insulin resistance [5, 6]. For instance, a study employing a multi-omics approach found that individuals with IR exhibited higher levels of fecal monosaccharides, which correlated with increased inflammatory cytokines [7, 8]. Moreover, certain gut bacteria, such as those belonging to the Lachnospiraceae family, were positively associated with IR, while others, like Bacteroidales, were linked to improved insulin sensitivity [9, 10]. These findings suggest that targeting specific gut microbial populations could offer novel therapeutic strategies for managing insulin resistance and its associated metabolic disorders [11-13].

Research indicates that a combination of genetic, autoimmune, and environmental factors contributes to the destruction of pancreatic cells. In limited cases (less than 10%), none of these factors are present in the patient, which classifies it as idiopathic [14]. In the aforementioned disease, the onset of cell destruction is usually sudden and can occur at any age; however, it predominantly starts in early childhood [1]. The younger the age of onset, the more severe the diabetes tends to be [15]. T1D accounts for 5 to 10% of all diabetes cases worldwide and is the most common type of diabetes in children and adolescents, with its incidence and prevalence increasing globally [16]. It is estimated that its prevalence will rise by 3% annually. Currently, about 542,000 people are living with T1D globally, with the highest numbers found in the United States, Brazil, India, and China [1]. A meta-analysis conducted in 2020 reported an incidence of 15 cases per 100,000 people and a prevalence of 9.5% worldwide [16]. Additionally, another study observed that the prevalence of T1D among individuals under the age of 19 in the United States increased from 1.48 per 1,000 people in 2001 to 2.15 per 1,000 people in 2017, with a total of 7,759 cases reported that year [17].

In Europe, it has been observed that the annual growth of the incidence of T1D is about 3.4%, which has doubled over the past 20 years [18]. Additionally, an extensive study conducted by the EURODIAB association in 17 European countries over 15 years (1989 to 2003) recorded more than 29,000 new cases of T1D, with the highest annual growth rate occurring in the age group under 5 years. This study predicted that if the current trend continues, new cases of diabetes in this age group (below 5 years) will double between 2005 and 2020, estimating that more than 24,000 new cases of T1D would occur in 2020 alone [19]. Regarding the prevalence of T1D in Iran, there is insufficient epidemiological information [20, 21]. However, a study conducted in Mazandaran province estimated the regional prevalence of this disease to be 0.04%, or 4.8 people per 100,000, in 2018 [21]. Furthermore, another study conducted in Fars's province in 2005 reported its incidence as 3.7 per 100,000 people per year [22].

Diabetes is a multifactorial disease caused by genetic, autoimmune, and environmental factors [15]. Additionally, non-environmental causes, such as gender and age, contribute to its development [23]. Surveys conducted over the last 50 years have shown that individuals with a low-risk genetic background are now developing this disease at a younger age, highlighting the increasing role of environmental factors [24, 25]. Given the significant impact of environmental factors on the development of T1D, various studies have investigated these influences. The most examined causes include the consumption of cow's milk during infancy, duration of breastfeeding, use of powdered milk in infancy, birth weight, and growth during the first months of life, vitamin D supplementation, age at which complementary feeding begins, infections especially enteroviruses and congenital measles and psychological stress [26, 27]. Other studies have explored additional influencing factors, such as maternal excess weight during pregnancy, first-degree relatives with diabetes [28], mode of delivery [29], environmental chemical pollutants, weather conditions [30], geographical factors, and the season and month of birth [31].

One significant gap between this study and previous studies is the limited exploration of environmental factors that may influence T1D onset, particularly maternal health, dietary practices, and socio-economic conditions. While existing studies have documented the prevalence and genetic predispositions associated with T1D, they often lack a comprehensive analysis of how local environmental influences, such as breastfeeding duration, introduction of cow's milk, and maternal weight gain, interact with genetic factors to affect disease risk. This oversight limits the understanding of T1D's multifactorial nature in southern Iran and calls for more targeted research. Since T1D is an autoimmune disease with multifactorial etiology, understanding the specific risk factors in this region is crucial for developing targeted prevention strategies. The study aims to fill this gap by identifying and analyzing various risk factors, including maternal health during pregnancy, infant feeding practices, and family history of autoimmune diseases, which may contribute to T1D onset in children and adolescents. Moreover, existing literature often presents a generalized view of T1D risk factors without considering regional variations that may significantly impact disease prevalence and manifestation. For example, while studies have identified certain environmental triggers like early childhood diet and infections as contributing factors to T1D, there is insufficient data on how these factors interact within southern Iran's cultural and socio-economic context.

Considering the rising number of T1D cases reported in the pediatric departments of Jiroft University of Medical Sciences hospitals, there is a pressing need to address this issue. The global increase in T1D incidence highlights the importance of preventive measures, particularly in children, to mitigate complications associated with the disease. Additionally, environmental factors play a crucial role in the development of T1D, prompting the need for a focused examination of these risk factors in affected children. This study aims to identify and analyze key risk factors related to T1D among children in the southern region of Kerman province.

## 2 MATERIALS AND METHODS

This study is a case-control study conducted in 2022 in the south of Iran. The statistical population included a case group consisting of children with T1D under 18 years of age who had a history of hospitalization or referral to the medical centers of Jiroft University of Medical Sciences. The control group comprised healthy children under 18 years old attending schools, preschools, and kindergartens in the region, as well as children referred to general surgery and orthopedic departments (without diabetes or related complications). Data collection commenced after obtaining ethical approval from Jiroft University of Medical Sciences. Patients were selected using a convenient sampling method based on the inclusion and exclusion criteria and their willingness to participate in the research. After explaining the goals and methods of the study to the parents of the children, questionnaires were administered and completed. For each patient in the case group, a patient of the same sex and similar age was selected as a control, and after obtaining their consent, the questionnaire was completed. The final sample size was determined based on the results of a pilot study involving 10 individuals from each group, utilizing a formula for comparing two means with a confidence level of 95% and a power of 90%. According to this formula, the initial sample size was estimated to be 78 individuals in each group.


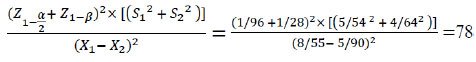


X1 = 8.55 (average anxiety of the intervention group)

S1 = 5.54 (standard deviation of the intervention group's anxiety)

X2 = 5.90 (average anxiety of the control group)

S2 = 4.64 (standard deviation of the control group's anxiety)

Z1-α/2 = 1.96 (confidence factor for 95%)

Z1-β = 1.28 (test power for 90%)

78 = Minimum sample size in each group

The criteria for inclusion in the study for the case group were: 1) having T1D in children and adolescents, confirmed by family reports, documentation, and a doctor's confirmation; 2) being under 18 years of age; and 3) willingness of patients and their families to participate in the study. For the control group, the inclusion criteria were: 1) not having any type of diabetes or glucose intolerance disorder; 2) being under 18 years of age; and 3) willingness to participate in the study. The exclusion criteria included any unwillingness to cooperate at any stage of the research. The questionnaire used in this research was developed based on potential risk factors related to T1D, informed by previous studies. It included sections with questions related to the child, as well as their mother, father, and siblings. This questionnaire was completed for subjects in both the case and control groups. To ensure its validity, input from pediatric endocrinology professors was sought, and after considering their feedback, the questionnaire was confirmed for its form and content. To assess reliability, the questionnaire was initially administered to 10 individuals from each group, yielding a Cronbach's alpha coefficient of 0.87.

Finally, data related to 158 children and adolescents were analyzed and divided into two groups of 79 individuals: case and control. The results were presented and summarized in the form of 14 tables, along with two sections for descriptive and inferential statistics. The collected data were entered into SPSS statistical software for analysis. To describe the data, descriptive statistics indicators, such as frequency, percentage, minimum and maximum values, mean, and standard deviation were utilized. For comparing quantitative variables with a normal distribution, an independent t-test was employed; for quantitative data with a non-normal distribution, the Mann-Whitney test was used; and for comparing qualitative data, the chi-square test was applied. Additionally, logistic regression analysis was conducted to calculate the odds ratios for variables influencing T1D. The significance level (*p*-value) in this study was set at 0.05.

## 3 RESULTS

In this study, 79 children and adolescents with T1D and 79 healthy children participated as the control group (Response Rate = 100%). The control group was matched with the case group based on age and gender. The average age in both groups was 10 ± 4 years, with the percentage of girls being 63.3% in the case group and 60.8% in the control group. None of the participants had ever contracted measles. Additionally, 44.3% of the patients were born in spring. Most participants in both groups were born at a gestational age of 9 months or more (81% in the case group and 84.8% in the control group). The weight distribution showed that 39.2% of the affected group and 20.3% of the control group were below optimal weight, while 69.69% and 43% were at optimal weight, respectively. Furthermore, 17.7% of the affected group and 10.1% of the control group were above optimal weight. Most mothers in both groups gained less than 11 kg during pregnancy, accounting for 54.4% in the case group and 72.2% in the control group. The predominant delivery methods for both groups were reported as 63.3% and 84.8%, respectively. In the affected group, the primary symptoms leading to diagnosis included diabetic ketoacidosis (DKA) in 27.8% of cases, nausea, vomiting, and abdominal pain in 21.5%, and polydipsia and polyuria in 50.7%, either alone or accompanied by lethargy and weight loss. Additionally, 54.4% of those affected experienced DKA during the course of their illness, as shown in Table **1**.

Table **2** shows the mean and standard deviation of quantitative variables in the case group. According to the results of the table, the average age is 10 ± 4 years, the average age at diagnosis is 7.1 ± 3.2 years, the average birth weight is 3.12 ± 0.54 kg, the average age of starting kindergarten or school is 5.9 ± 1.3 years, the average age of obtaining a smartphone is 6.5 ± 3.4 years, the average age of the mother at the time of childbirth is 27.4 ± 6.4 years, the average age of the father at the time of childbirth is 31 ± 7.3 years, and the average age of siblings with T1D at diagnosis is 6.5 ± 2.5 years.

Table **3** shows the mean and standard deviation of the quantitative variables in the control group. According to the results of the table, the average age is 10 ± 4 years, the average birth weight is 3.22 ± 0.40 kg, the average age of starting kindergarten or school is 5.5 ± 1.3 years, the average age of obtaining a smartphone is 5.8 ± 3.3 years, the average age of the mother at childbirth is 28.4 ± 1.5 years, the average age of the father at childbirth is 31.7 ± 5.8 years, and the average age of siblings with T1D at diagnosis is 4 ± 0 years.

Table **4** shows the number and frequency of qualitative variables related to nutritional factors in the case and control groups. According to the results of the table, regarding the consumption of powdered milk in the first six months of life, most participants in both groups reported “no” (67.1% in the case group and 79.7% in the control group). For powdered milk consumption before the age of one year, most also responded “no” (69.6% in the case group and 74.7% in the control group). Concerning the use of cow's milk before the age of one year, 57% of the case group reported “yes.” In contrast, 75.9% of the control group indicated “no” regarding cow's milk consumption before one year. Regarding vitamin D supplementation, most participants in both groups answered “yes” (54.4% in the case group and 77.2% in the control group).

Table **5** shows the mean and standard deviation of quantitative variables of nutritional factors in the case group. According to the results, the average age of starting complementary feeding is 4.2 ± 1.6 months. Average duration of breastfeeding is 16.8 ± 7.2 months.

Table **6** shows the mean and standard deviation of quantitative variables of nutritional factors in the control group. According to the results of the table, the average age of starting complementary feeding is 5.2 ± 1 months, and the average duration of breastfeeding is 19.2 ± 5.7 months.

Table **7** presents the number and frequency of qualitative variables related to family genetic risk factors in both the case and control groups. The data indicates that the prevalence of autoimmune diseases, excluding diabetes, is 19% in the case group and 5.1% in the control group. Among the affected individuals, thyroid disorders were the most common autoimmune disease, observed in 6 participants (7.6%), followed by celiac disease in 5 participants (6.3%). One individual had both thyroid and celiac diseases simultaneously (1.3%). Additionally, 2 participants (2.6%) had vitiligo, and one participant (1.3%) was diagnosed with rheumatism. In contrast, the control group reported 3 cases (3.8%) of thyroid disorders and one case (1.3%) of vitiligo.

The prevalence of chronic diseases other than autoimmune conditions was found to be 17.7% in the case group and 10.1% in the control group. The incidence of T1D among mothers was recorded at 10.1% for the case group compared to 2.5% for the control group, while T2D prevalence among mothers was noted at 1.5% for cases and 3.8% for controls. Furthermore, infertility issues among mothers were reported at rates of 1.5% and 1.3%, respectively, along with a significant difference in the use of medications during pregnancy: 21.5% in the case group *versus* 5.1% in the control group. Notably, among these cases, one individual used a steroid medication while another used levothyroxine; all other cases involved insulin usage.

The previous prevalence of autoimmune diseases in mothers, excluding diabetes, was reported at 26.6% in the affected group and 12.7% in the control group. In the affected group, 6 individuals (7.6%) had thyroid disorders, 5 (6.3%) experienced stillbirths, 4 (1.5%) had rheumatism, 2 (2.5%) had goiter, 2 (2.5%) had multiple sclerosis (MS), and 1 individual (1.3%) had both thyroid disorder and rheumatic disease, while another individual (1.3%) had both thyroid disorder and stillbirth. In the control group, 7 individuals (8.9%) had thyroid disorders and 3 (3.8%) had MS. The prevalence of type T1D in mothers' families was found to be 7.6% in the affected group and 6.3% in the control group, while the prevalence of T2D in mothers' families was reported as 22.8% and 24.1%, respectively. The prevalence of T1D among fathers was noted at 8.9% in the affected group and 0% in the control group, with T2D prevalence among fathers at 6.3% and 7.6%, respectively. Additionally, the prevalence of autoimmune diseases in fathers, excluding diabetes, was reported as 10.1% for the affected group and 3.8% for the control group. In the case group, there were 3 patients (3.3%) with psoriasis and one individual (1.3%) with a combination of thyroid disorder, ulcerative colitis, Crohn's disease, goiter, and rheumatism. In the control group, one person (1.3%) had a combination of thyroid disorder, psoriasis, celiac disease, and ulcerative colitis. The prevalence of T1D among fathers' families was found to be 19% in the affected group and 3.8% in the control group, while T2D prevalence among fathers' families was reported as 32.9% and 17.7%, respectively. The prevalence of T1D among siblings was recorded at 16.7% in the affected group and 1.3% in the control group; T2D prevalence among siblings was noted at 1.3% for one sibling and 0% for another. The prevalence of autoimmune diseases other than diabetes among siblings was found to be 21.5% in the affected group compared to 5.1% in the control group; specifically, in the affected group, there were 6 individuals (7.6%) with both thyroid disorders and celiac disease, along with 2 individuals (2.5%) having psoriasis, and one individual (1.3%) with vitiligo and rheumatic diseases. In contrast, the control group reported one individual (1.3%) with thyroid disorders as well as celiac disease and rheumatic diseases.

Table **8** shows the average difference of quantitative variables in the case and control groups. Independent t-tests and Mann-Whitney tests (for the variables of fetal age at birth and age of going to kindergarten or school due to their non-normal distribution) were used to assess the difference in means. According to the results of the table, the difference in the average age of going to kindergarten or school is significant (*p* = 0.015). The average for the control group is 5.45 ± 1.27 years for the age of going to kindergarten or school.

Table **9** shows the difference in the frequency distribution of qualitative demographic variables in the case and control groups. The chi-square test was used to assess the differences in frequency distribution. According to the results of the table, there are significant differences in the following variables: weight at the end of one year (*p* = 0.003), weight at the end of 18 months (*p* = 0.002), maternal weight gain during pregnancy (*p* = 0.021), with a higher frequency of 11 kg or more in the case group, and the type of delivery (*p* = 0.002), with a greater frequency of cesarean sections in the case group.

Table **10** shows the average difference in quantitative variables related to nutritional factors in the case and control groups. The Mann-Whitney test was used to assess the differences in means due to the non-normal distribution of the data. According to the results of the table, the difference in the average age of starting complementary feeding is significant (*p* = 0.000), with the average for the control group being 5.23 ± 0.99 months for the age at which complementary feeding begins. Additionally, the difference in the average duration of breastfeeding is significant (*p* = 0.030), with the average for the control group being 19.20 ± 5.72 months for the duration of breastfeeding.

Table **11** shows the difference in the frequency distribution of qualitative variables related to nutritional factors in the case and control groups. The chi-square test was used to assess the differences in frequency distribution. According to the results of the table, there is a significant difference regarding the consumption of cow's milk before the age of one year (*p* = 0.000), with a higher frequency of consumption in the case group. Additionally, there is a significant difference in the consumption of vitamin D supplements (*p* = 0.003), with a higher frequency of consumption in the control group.

Table **12** shows the difference in the frequency distribution of qualitative variables related to family genetic risk factors in the case and control groups. The chi-square test was used to assess the differences in frequency distribution. According to the results of the table, there is a significant difference regarding autoimmune diseases other than diabetes (*p* = 0.007), with a higher prevalence in the case group. Additionally, the use of specific medications during pregnancy showed a significant difference (*p* = 0.002), with a higher prevalence in the case group. The history of autoimmune disease in mothers, excluding diabetes, also demonstrated a significant difference (*p* = 0.028), with a higher frequency in the case group. Furthermore, the prevalence of T1D in fathers was higher in the case group, as was the history of T1D in fathers' families (*p* = 0.003), indicating that this history was more common in the case group. The prevalence of T2D in fathers' families was also higher in the case group. Among siblings, there was a significant difference regarding autoimmune diseases other than diabetes (*p* = 0.002), with a higher frequency of such diseases in the case group. Additionally, the prevalence of T1D among siblings was greater (*p* = 0.035) in the case group, indicating significant differences between both groups.

Table **13** presents the odds ratio, confidence interval, significance level, and regression coefficient of intervening factors related to T1D in the studied community, focusing on the individual impact of each variable. The results indicate that the variables concerning the age of entering kindergarten or school and maternal T1D did not show significant effects due to their lack of statistical significance. In contrast, starting complementary feeding at an older age, longer breastfeeding duration, and vitamin D supplementation were found to have a protective effect against T1D. The statistical analysis for paternal T1D could not be accurately calculated due to zero frequency in the control group. Additionally, other variables significantly increased the risk of developing diabetes.

Table **14** displays the odds ratio, confidence interval, significance level, and regression coefficient of intervening factors related to T1D in the studied community, examining the simultaneous impact of various variables. The results indicate that none of the variables had a significant effect, except for the consumption of cow's milk before the age of one year, which was associated with a notable increase in the risk of T1D in the logistic regression analysis, both individually and in combination with other factors.

## 4 DISCUSSION

The objective of the present study was to identify factors linked to T1D in the southern region of Kerman province in 2022. The results indicated that 63.3% of individuals diagnosed with T1D were girls, with an average age of diagnosis at 7.1 ± 3.2 years. A majority of the cases were born in the spring and summer months (69.6%). The most frequently reported symptom prior to diagnosis was polyuria and excessive thirst, experienced by 50.7% of patients, while DKA was the first symptom noted in 27.8% of cases. The total incidence of DKA among patients was recorded at 54.4%, with girls experiencing DKA more often than boys. In a study by Al-Ghamdi and Fureeh (2018), T1D was also found to be more common in girls, with 40.8% of patients presenting to medical centers for the first time due to DKA; this figure was lower than what was observed in the current research [32].

In the study conducted by Maahs DM *et al.* (2010), it was reported that in most regions, there was little difference in the prevalence of T1D between boys and girls; however, in areas with lower incidence rates, a higher percentage of girls was observed [2]. The research by Çiçekli *et al.* (2022) indicated an average age of diagnosis of 6.30 ± 4.03 years, which is comparable to the findings of this study [33]. Vakili and Farivar (2002) also noted that most patients were born in the spring and summer, aligning with the results obtained in this research [31]. According to Demir F and colleagues (2015), the majority of patients were diagnosed between the ages of 6 and 8, with polyuria and excessive thirst being the primary symptoms; additionally, DKA was more prevalent among girls [34]. Lah Tomulić *et al.* (2022) found that approximately a quarter of patients presented with DKA as their primary symptom, which is similar to the percentage reported in this study [35]. Conversely, Szypowska *et al.* (2017) reported an overall incidence of DKA among patients in Poland at 28.6%, which is lower than the findings from this study [36]. This discrepancy may be attributed to insufficient healthcare facilities and the financial hardships faced by many diabetic patients in this region, leading to a greater incidence of DKA due to limited access to insulin.

In this research, significant differences were found among the investigated demographics, including the age of starting kindergarten, weight at one year and 18 months, maternal weight gain, and delivery method between the case and control groups. When examining the impact of these factors on the likelihood of developing T1D, it was noted that unfavorable weight at one year and 18 months (three times), maternal weight gain of 11 kg or more during pregnancy (1.2 times), and cesarean delivery (2.3 times) all increased the risk of diabetes. However, in the simultaneous analysis of these variables' effects on T1D, none showed significant results. According to Magnus *et al.* (2015), weight gain at one year was associated with an increased risk of T1D [37]. Similarly, Waernbaum *et al.* (2019) and Magnus *et al.* (2018) found that maternal weight gain could elevate the risk of T1D in children; however, maternal overweight status during pregnancy did not significantly contribute to the development of this disease [38, 39]. Additionally, Tanoey *et al.* (2019) and Begum *et al.* (2019) reported that cesarean delivery might increase the risk of T1D [29, 40]. Conversely, Khashan *et al.* (2014) concluded that cesarean sections did not influence the incidence of diabetes in children [41].

In this research, the findings indicated that delaying the start of complementary feeding by each month, extending the duration of breastfeeding by each month, and taking vitamin D supplements were associated with a reduced risk of T1D, with odds ratios of 0.56, 0.94, and 0.35, respectively. However, when examining the simultaneous effects of these variables on T1D, none showed a significant impact. The consumption of cow's milk before the age of one year was the only factor that increased the risk of T1D, with an odds ratio of 1.197, whether considered alone or in combination with other factors. Additionally, there was no significant difference in powdered milk consumption between the case and control groups, possibly due to lower consumption rates in the studied population resulting from financial constraints. The study by Çiçekli *et al.* (2022) also concluded that each month of breastfeeding reduces the risk of developing diabetes by a factor of 0.83 and starting complementary feeding before six months increases the risk of T1D [33]. In this study, most diabetic children began complementary feeding at less than six months and were breastfed for a shorter duration compared to non-diabetic children.

In the study by Dong *et al.* (2013), it was found that early vitamin D supplementation can lower the risk of diabetes [42]. The current research also indicates that non-diabetic children are more likely to use vitamin D supplements compared to their diabetic counterparts. Additionally, Chia JSJ *et al.* (2017) identified cow's milk as a significant nutritional risk factor for T1D [43]. This study concluded that many diabetic patients consumed cow's milk before their first birthday, potentially due to financial constraints limiting access to powdered milk or a lack of adequate information on proper infant feeding practices.

Another finding from this study regarding genetic and familial factors influencing T1D is that families with a history of T1D in the father or siblings, as well as the presence of type 1 or type 2 diabetes and autoimmune diseases in the paternal family, exhibited a higher risk and prevalence of T1D. These results highlight the significant role of genetics in this condition. The findings from the current study emphasize the considerable impact of genetic and familial factors on the likelihood of developing T1D. Families with a history of T1D, especially when the father or siblings are affected, demonstrate a notably higher prevalence of the disease. This reinforces the critical importance of genetic predisposition in T1D development. The genetic linkage underscores family history's role as a predictive factor for the disease.

Furthermore, the presence of other autoimmune diseases within the paternal family increases the risk of T1D. This relationship indicates that shared genetic factors may predispose individuals to T1D as well as other autoimmune conditions. The results of this study are consistent with existing literature that identifies familial autoimmune diseases as significant contributors to T1D susceptibility. In line with this finding, Çiçekli *et al.* (2022) also noted that a family history of T1D increases the risk for children [33], while Dana Siadat *et al.* found it contributes to a higher incidence of T1D in children [44]. Additionally, Algert *et al.* (2009) indicated that maternal T1D can significantly impact the child's risk of developing the disease [45]. However, the current study did not find a significant relationship between maternal T1D and children's diabetes; it is possible that a larger sample size could reveal a significant association.

In the current study, it was found that autoimmune diseases were more prevalent among children with T1D, particularly thyroid disorders and celiac disease, which were more common in girls. Głowińska *et al.* (2020) reported a simultaneous increase in the incidence of autoimmune diseases and T1D in children and adolescents [46]. Similarly, Burbaud *et al.* (2022) identified Hashimoto's disease and celiac disease as the most common autoimmune conditions associated with T1D in this demographic [47]. The findings suggest a significant relationship between T1D and the presence of autoimmune disorders, highlighting the need for ongoing monitoring and management of these coexisting conditions.

### CONCLUSION

The findings of this study indicate that various environmental factors, including nutritional aspects, such as the consumption of cow's milk before the age of one, the use of vitamin D supplements, the timing of complementary feeding, and the duration of breastfeeding, can increase the risk of T1D in children. Additionally, it was observed that the prevalence of T1D is higher in children with a family history of type 1 or T2D or autoimmune diseases. Given that the incidence of diabetes is rising in the southern region of Kerman province as well as in other areas, addressing these environmental factors could significantly contribute to preventing and reducing the prevalence of T1D and its complications.

### STUDY LIMITATIONS

Several limitations affected this study. Some patients were excluded from the analysis due to incomplete information in their files. The statistical population of the study consisted of a case group, which included children with T1D under 18 years of age who had been hospitalized or referred to medical centers at Jiroft University of Medical Sciences, and a control group comprising healthy children under 18 years old attending schools, preschools, and kindergartens in the region, as well as children referred to general surgery and orthopedic departments (without diabetes or related complications). Consequently, caution should be exercised when generalizing the findings. Given these limitations, it is recommended that future studies involve a larger sample size and be conducted across different hospitals to enhance the reliability of the results. Another limitation of the present study is the lack of body mass index (BMI) calculations for the children involved. Therefore, it is suggested that future researchers incorporate BMI as a variable in their studies.

## Figures and Tables

**Table 1:** Number and percentage of frequency of qualitative demographic variables.

**Variable**	**Class**	**In the Case Group**	**The Percentage of Frequency in the Case Group**	**In the Control Group**	**The Frequency Percentage in the Control Group**
Gender	Girl	50	63.3	48	60.8
	Boy	29	36.7	31	39.2
Birth rank	First	25	31.6	32	40.5
	Second	27	34.2	33	41.8
	Third and more	27	34.2	14	17.7
Birthday season	Spring	35	44.3	23	29.1
	Summer	20	25.3	28	35.4
	Fall	9	11.4	18	22.8
	Winter	15	19	10	12.7
Fetal age at birth	Less than 9 months	15	19	12	15.2
	9 months and more	64	81	67	84.8
The weight at the end of one year	Less than optimal	31	39.2	16	20.3
	Optimal	34	43	55	69.6
	More than optimal	14	17.7	8	10.1
Weight at the end of 18 months	Less than optimal	33	41.8	17	21.5
	Optimal	32	40.5	54	68.4
	More than optimal	14	17.7	8	10.1
The mother's weight gain during pregnancy	Less than 11 kg	43	54.4	57	72.2
	11 Kg and More	36	45.6	22	27.8
Type of childbirth	Natural	50	63.3	67	84.8
	Cesarean section	29	36.7	12	15.2
Early symptoms of diabetes	Hyperdrinking and hyperuricemia	13	16.5	0	0
	Nausea, vomiting, and heartache	17	21.5	0	0
	Drunk and lethargic	14	17.8	0	0
	Binge drinking and weight loss	13	16.5	0	0
	DKA	22	27.8	0	0
Incidence of DKA	Yes	43	54.4	-	-
	No	36	45.6	-	-

**Table 2:** Mean and standard deviation of quantitative demographic variables in the case group.

**Variable**	**Number**	**Minimum**	**Maximum**	**Mean ± Standard Deviation (SD)**
Age	79	3	18	10 ± 4
Age at diagnosis	79	2	15	7.1 ± 3.2
Birth weight	79	1.8	4.2	3.12 ± 0.54
The age of going to kindergarten or school	68	2	8	5.9 ± 1.3
The starting age of owning a smartphone	75	2	14	6.5 ± 3.4
Mother's age at the time of motherhood	79	16	51	27.4 ±6.4
Father's age at the time of fatherhood	79	20	55	31 ± 7.3
Age of siblings with T1D at the time of diagnosis	13	2	10	6.5 ±2.5

**Table 3:** Mean and standard deviation of quantitative demographic variables in the control group.

**Variable**	**Number**	**Minimum**	**Maximum**	**Mean** ± **SD**
Age	79	3	18	10 ± 4
Birth weight	79	2.2	4.3	3.22 ±0.4
The age of going to kindergarten or school	62	2.2	7	5.5 ± 1.3
The starting age of owning a smartphone	75	1	14	5.8 ± 3.3
Mother's age at the time of motherhood	79	20	42	28.4 ± 5.1
Father's age at the time of fatherhood	79	22	50	31.7 ± 5.8
Age of siblings with T1D at the time of diagnosis	1	4	4	4 ± 0

**Table 4:** Number and frequency percentage of qualitative variables of nutritional factors in case and control groups.

**Variable**	**Class**	**Number in the Case Group**	**The Percentage of Frequency in the Case Group**	**The Number In the Control Group**	**The Frequency Percentage in the Control Group**
Use of powdered milk before the age of six months	Yes	26	32.9	16	20.3
	No	53	67.1	63	79.7
Use of powdered milk before one year of age	Yes	24	30.4	20	25.3
	No	55	69.6	59	74.7
Consumption of cow's milk before one year of age	Yes	45	57	19	24.1
	No	34	43	60	75.9
Taking vitamin D supplements	Yes	43	54.4	61	77.2
	No	36	45.6	18	22.8

**Table 5:** Mean and standard deviation of quantitative variables of nutritional factors in the case group.

**Variable**	**Number**	**Minimum**	**Maximum**	**Mean** ± **SD**
The age of starting complementary food	79	1	6	4.2 ± 1.6
Duration of breast milk consumption	79	0	32	16.8± 7.2

**Table 6:** Mean and standard deviation of quantitative variables of nutritional factors in the case group.

**Variable**	**Number**	**Minimum**	**Maximum**	**Mean** ± **SD**
The age of starting complementary food	79	3	6	5.2 ±1
Duration of breast milk consumption	79	8	42	19.2 ±5.7

**Table 7:** Number and frequency percentage of qualitative variables of family genetic risk factors in case and control groups.

**Variable**	**Class**	**Number in the Case Group**	**The Percentage of Frequency in the Case Group**	**The Number in the Control Group**	**The Frequency Percentage in the Control Group**
Autoimmune diseases (other than diabetes)	Yes	15	19	4	5.1
	No	64	81	75	94.9
Chronic diseases (other than diabetes)	Yes	14	17.7	8	10.1
	No	45	82.3	71	89.9
The mother has T1D	Yes	8	10.1	2	2.5
	No	71	89.9	77	97.5
The mother has T2D	Yes	4	5.1	3	3.8
	No	75	94.9	76	96.2
Mother suffering from gestational diabetes	Yes	13	16.5	7	8.9
	No	66	83,5	72	91.1
The problem of infertility in the mother and the use of ovarian stimulation medication	Yes	4	5.1	1	1.3
	No	75	94.9	78	98.7
Use of certain drugs during pregnancy	Yes	17	21.5	3	5.1
	No	62	78.5	75	94.9
A history of autoimmune disease in the mother other than diabetes	Yes	21	26.6	10	12.7
	No	58	73.4	69	87.3
A history of T1D in the mother's family	Yes	6	7.6	5	6.3
	No	73	92.4	74	93.7
History of T2D in mother's family	Yes	18	22.8	19	24.1
	No	61	77.2	60	75.9
Father has T1D	Yes	7	8.9	0	0
	No	72	91.1	79	100
Father has T2D	Yes	5	6.3	6	7.6
	No	74	93.7	73	92.4
A history of autoimmune disease in the father, other than diabetes	Yes	8	10.1	3	3.8
	No	71	89.9	76	96.2
A history of T1D in the father's family	Yes	15	19	3	3.8
	No	64	81	76	96.2
A history of T2D in the father's family	Yes	26	32.9	14	17.7
	No	53	67.1	65	82.3
Siblings suffering from autoimmune diseases other than diabetes	Yes	17	21.5	4	5.1
	No	62	78.5	75	94.9
Siblings suffering from T1D	Yes	13	16.7	1	1.3
	No	66	83.3	77	98.7
Siblings suffering from T2D	Yes	1	1.3	0	0
	No	78	98.7	79	100

**Table 8:** Examining the average difference of quantitative demographic variables in case and control groups.

**Variable**	**Mean** ± **SD in the Case Group**	**Mean** ± **SD in the Control Group**	**Test Statistic (Degrees of Freedom) or Z and U- Man-Whitney**	***p*-value**
Birth weight	3.12 ± 0.54	3.22 ± 0.40	-1.309 (144.75)	0.193
Age of mothers at the time of birth	27.41 ± 6.42	28.39 ± 5.06	-0.058 (156)	0.292
Age of fathers at the time of birth	30.97 ± 7.34	31.68 ± 5.84	-4.671 (148.51)	0.502
The age of going to kindergarten or school	5.19 ± 1.34	5.45 ± 1.27	-2.435 (1613.00)	0.015
The starting age of owning a smartphone	6.50 ± 3.37	5.76 ± 3.28	1.356 (147)	0.177

**Table 9:** Examining the difference in the frequency distribution of qualitative demographic variables in case and control groups.

**Variable**	**Class**	**Frequency (Percentage) in the Case Group**	**Frequency (Percentage) in the Control Group**	**The Result of the Chi-square Test**
Gender	Girl	50 (63.3%)	48 (60.8%)	*p* = 0.743
	Boy	29 (36.7%)	31 (39.2%)	
Birth rank	First	25 (31.6%)	32 (40.5%)	*p* = 0.061
	Second	27 (34.2%)	33 (41.8%)	
	Third and more	27 (34.2%)	14 (17.7%)	
Birthday season	Spring	35 (44.3%)	23 (29.1%)	*p* = 0.051
	Summer	20 (25.3%)	28 (35.4%)	
	Fall	9 (11.4%)	18 (22.8%)	
	Winter	15 (19.0%)	10 (12.7%)	
Fetal age at birth	Less than nine months	15 (19.0%)	12 (15.4%)	*p* = 0.550
	Nine months or more	64 (81.0%)	67 (84.6%)	
The weight at the end of one year	Less than optimal	31 (39.2%)	16 (20.3%)	*p* = 0.003
	Optimal	34 (43.0%)	55 (69.6%)	
	More than optimal	14 (17.7%)	8 (10.1%)	
Weight at the end of 18 months	Less than optimal	33 (41.8%)	17 (21.5%)	*p* = 0.002
	Optimal	32 (40.5%)	54 (68.4%)	
	More than optimal	14 (17.7%)	8 (10.1%)	
The mother's weight gain during pregnancy	Less than 11 kg	43 (54.4%)	57 (72.2%)	*p* = 0.021
	11 kg and more	36 (45.6%)	22 (27.8%)	
Type of childbirth	Natural	50 (63.3%)	67 (84.8%)	*p* = 0.002
	Cesarean Section	29 (36.7%)	12 (15.2%)	

**Table 10:** Examining the average difference of the quantitative variables of nutritional factors in case and control groups.

**Variable**	**Mean ± SD in the Case Group**	**Mean ± SD in the Control Group**	**Z and U Mann-Whitney test**	***p*-value**
The age of starting complementary food	4.24 ± 1.57	5.23 ± 0.99	-4.095 (1999.500)	0.000
Duration of breast milk consumption	16.84 ± 7.20	19.20 ± 5.72	1.875 (2588.000)	0.030

**Table 11:** Examining the difference in the frequency distribution of qualitative variables of nutritional factors in case and control groups.

**Variable**	**Class**	**Frequency (Percentage) in the Case Group**	**Frequency (Percentage) in the Control Group**	**Chi-square Test Result**
Use of powdered milk before the age of six months	Yes	26 (32.9%)	16 (20.3%)	*p* = 0.072
	No	53 (67.1%)	63 (79.7%)	
Use of powdered milk before one year of age	Yes	24 (30.4%)	20 (25.3%)	*p* = 0.478
	No	55 (69.6%)	59 (74.7%)	
Consumption of cow's milk before one year of age	Yes	45 (57.0%)	19 (24.1%)	*p* = 0.000
	No	34 (43.0%)	60 (75.9%)	
Taking vitamin D supplements	Yes	43 (54.4%)	61 (77.2%)	*p* = 0.003
	No	36 (45.6%)	18 (22.8%)	

**Table 12:** Examining the difference in the frequency distribution of qualitative variables of family genetic risk factors in case and control groups.

**Variable**	**Class**	**Frequency (Percentage) in the Case Group**	**Frequency (Percentage) in the Control Group**	**Chi-square Test Result**
Autoimmune diseases (other than diabetes)	Yes	15 (19%)	4 (5.1%)	*p* = 0.007
	No	64 (81%)	75 (94.9%)	
Chronic diseases (other than diabetes)	Yes	14 (17.7%)	8 (10.1%)	*p* = 0.107
	No	45 (82.3%)	71 (89.9%)	
The mother has T1D	Yes	8 (10.1%)	2 (2.5%)	*p* = 0.050
	No	71 (89.9%)	77 (97.5%)	
The mother has T2D	Yes	4 (5.1%)	3 (3.8%)	*p* = 0.699
	No	75 (94.9%)	76 (96.2%)	
Mother suffering from gestational diabetes	Yes	13 (16.5%)	7 (8.9%)	*p* = 0.151
	No	66 (83.5%)	72 (91.1%)	
The problem of infertility in the mother and the use of ovarian stimulation medication	Yes	4 (5.1%)	1 (1.3%)	*p* = 0.173
	No	75 (94.9%)	78 (98.7%)	
Use of special medicine during pregnancy	Yes	17 (21.5%)	3 (5.1%)	*p* = 0.002
	No	62 (78.5%)	75 (94.9%)	
-	Yes	21 (26.6%)	10 (12.7%)	*p* = 0.028
	No	58 (73.4%)	69 (87.3%)	
A history of autoimmune disease in the mother other than diabetes	Yes	6 (7.6%)	5 (6.3%)	*p* = 0.755
	No	73 (92.4%)	74 (93.7%)	
A history of T1D in the mother's family	Yes	18 (22.8%)	19 (24.1%)	*p* = 0.851
	No	61 (77.2%)	60 (75.9%)	
History of T2D in mother's family	Yes	7 (8.9%)	0 (0%)	*p* = 0.007
	No	72 (91.1%)	79 (100%)	
Father has T1D	Yes	5 (6.3%)	6 (7.6%)	*p* = 0.755
	No	74 (93.7%)	73 (92.4%)	
Father has T2D	Yes	8 (10/1%)	3 (3/8%)	*p* = 0.118
	No	71 (89.9%)	76 (96.2%)	
A history of autoimmune disease in the father, other than diabetes	Yes	15 (19%)	3 (3.8%)	*p* = 0.003
	No	64 (81%)	76 (96.2%)	
A history of T1D in the father's family	Yes	26 (32.9%)	14 (17.7%)	*p* = 0.028
	No	53 (67.1%)	65 (82.3%)	
A history of T2D in the father's family	Yes	17 (21.5%)	4 (5.1%)	*p* = 0.002
	No	62 (78.5%)	75 (94.9%)	
Siblings suffering from autoimmune diseases other than diabetes	Yes	13 (16.7%)	1 (1.3%)	*p* = 0.001
	No	66 (83.3%)	77 (98.7%)	
T1D in siblings	Yes	1 (1.3%)	0 (0%)	*p* = 0.500
	No	78 (98.7%)	79 (100%)	

**Table 13:** presents the odds ratios, confidence intervals, significance levels, and regression coefficients for intervening factors related to T1D within the studied community. This table examines the simultaneous effects of various variables on the occurrence of T1D.

**Variable**	**Regression Coefficient**	**Standard Error**	**Odds Ratio**	**Confidence Interval**	***p*-value**
Kindergarten or school-age	0.270	0.138	1.309	1.718 - 0.998	0.052
Weight at the end of one year	1.110	0.334	3.033	5.836 - 1.576	0.001
Weight at the end of 18 months	1.155	0.333	3.172	6.096 - 1.651	0.001
Maternal weight gain during pregnancy	0.774	0.338	2.169	4.205 - 1.119	0.022
Type of delivery	1.175	0.391	3.238	6.699 - 1.505	0.003
Age of starting complementary feeding	-0.578	0.137	0.561	0.734 - 0.429	0.000
Duration of breast milk consumption	-0.057	0.026	0.945	0.994 - 0.898	0.027
Consumption of cow's milk before one year of age	1.430	0.348	4.180	8.263 - 2.114	0.000
Taking vitamin D supplements	-1.043	0.351	0.352	0.701 - 0.177	0.003
Autoimmune diseases (other than diabetes)	1.480	0.588	4.395	13.910 - 1.388	0.012
Use of certain drugs during pregnancy	1.637	0.582	5.141	16.074 - 1.644	0.005
History of autoimmune disease in mother other than diabetes	0.916	0.424	2.498	5.730-1.089	0/031
Father has T1D	2.129	1.519	1.772	0.999-0.000	0.000
History of T1D in the father's family	1.781	0.655	5.937	21.528 - 1.645	0.007
A history of T2D in the father's family	0.823	0.380	2.278	4.793 - 1.082	0.030
Siblings suffering from autoimmune diseases other than diabetes	1.637	0.582	5.141	16.074 - 1.644	0.005
T1D in siblings	2.734	1.051	15.4	120.897-1.962	0.009
The mother has T1D	1.467	0.808	4.338	21.118 - 0.891	0.069

**Table 14:** Odds ratio, confidence interval, significance level, and regression coefficient of intervening factors in T1D in the community under study in the form of examining the simultaneous effect of variables on T1D.

**Variable**	**Regression Coefficient**	**Standard Error**	**Odds Ratio**	**Confidence Interval**		***p*-value**
				**Lowest**	**Highest**	
Kindergarten or school-age	-0.182	0.285	0.834	0.503	1.383	0.481
Weight at the end of one year	-19.288	197.612	0.000	0.000	0.999	0.000
Weight at the end of 18 months	2.062	1.976	0.906	0.000	0.999	0.000
Maternal weight gain during pregnancy	1.003	0.610	2.725	0.824	0.012	0.100
Type of delivery	0.981	0.702	2.667	0.674	10.559	0.162
Age of starting complementary feeding	0.462	0.301	0.630	0.349	1.137	0.125
Duration of breast milk consumption	0.018	0.059	0.019	0.908	1.143	0.754
Consumption of cow's milk before one year of age	1.525	0.686	4.597	1.197	17.649	0.026
Taking vitamin D supplements	-0.068	0.688	0.934	0.242	3.599	0.921
Autoimmune diseases (other than diabetes)	1.508	0.942	4.519	1.713	28	0.109
Use of certain drugs during pregnancy	0.539	0.996	1.714	0.243	12	0.589
History of autoimmune disease in mother other than diabetes	0.459	0.751	1.582	0.363	6.900	0.541
Father has T1D	19.916	1.452	691.445	0.000	0.003	0.999
History of T1D in the father's family	1.764	1.019	5.836	0.791	43.038	0.084
A history of T2D in the father's family	0.881	0.648	2.414	0.678	8.593	0.174
Siblings suffering from autoimmune diseases other than diabetes	1.322	1.286	3.752	0.302	46.661	0.304
T1D in siblings	19.596	1.034	49.323	0.000	0.003	0.998
The mother has T1D	1.531	1.459	4.624	0.265	80.789	0.294

## Data Availability

All data generated or analyzed during this study are included in this published article.

## LIST OF ABBREVIATIONS

T1D: Type 1 Diabetes
T2D: Unlike in Type 2 Diabetes
IR: Insulin Resistance
BMI: Body Mass Index

## STANDARDS OF REPORTING

STROBE guidelines were followed.

## References

[1] Rak K., Bronkowska M. (2018). Immunomodulatory effect of vitamin D and its potential role in the prevention and treatment of type 1 diabetes mellitus—A narrative review.. Molecules.

[2] Maahs D.M., West N.A., Lawrence J.M., Mayer-Davis E.J. (2010). Epidemiology of type 1 diabetes.. Endocrinol. Metab. Clin. North Am..

[3] Li W.Z., Stirling K., Yang J.J., Zhang L. (2020). Gut microbiota and diabetes: From correlation to causality and mechanism.. World J. Diabetes.

[4] Shen J., Obin M.S., Zhao L. (2013). The gut microbiota, obesity and insulin resistance.. Mol. Aspects Med..

[5] Han J.L., Lin H.L. (2014). Intestinal microbiota and type 2 diabetes: From mechanism insights to therapeutic perspective.. World J. Gastroenterol..

[6] He C., Shan Y., Song W. (2015). Targeting gut microbiota as a possible therapy for diabetes.. Nutr. Res..

[7] Burcelin R., Serino M., Chabo C., Blasco-Baque V., Amar J. (2011). Gut microbiota and diabetes: from pathogenesis to therapeutic perspective.. Acta Diabetol..

[8] Zhao X., An X., Yang C., Sun W., Ji H., Lian F. (2023). The crucial role and mechanism of insulin resistance in metabolic disease.. Front. Endocrinol. (Lausanne).

[9] Most J., Goossens G.H., Reijnders D., Canfora E.E., Penders J., Blaak E.E. (2017). Gut microbiota composition strongly correlates to peripheral insulin sensitivity in obese men but not in women.. Benef. Microbes.

[10] Delzenne N.M., Cani P.D. (2011). Gut microbiota and the pathogenesis of insulin resistance.. Curr. Diab. Rep..

[11] Zhang X.Y., Chen J., Yi K., Peng L., Xie J., Gou X., Peng T., Tang L. (2020). Phlorizin ameliorates obesity-associated endotoxemia and insulin resistance in high-fat diet-fed mice by targeting the gut microbiota and intestinal barrier integrity.. Gut Microbes.

[12] Caricilli A., Saad M. (2013). The role of gut microbiota on insulin resistance.. Nutrients.

[13] Wastyk H.C., Fragiadakis G.K., Perelman D., Dahan D., Merrill B.D., Feiqiao B.Y. (2021). Gut-microbiota-targeted diets modulate human immune status.. Cell.

[14] PRISMA Flow Diagram.. https://www.prisma-statement.org/prisma-2020-flow-diagram.

[15] Craig M.E., Kim K.W., Isaacs S.R., Penno M.A., Hamilton-Williams E.E., Couper J.J., Rawlinson W.D. (2019). Early-life factors contributing to type 1 diabetes.. Diabetologia.

[16] Mobasseri M., Shirmohammadi M., Amiri T., Vahed N., Hosseini Fard H., Ghojazadeh M. (2020). Prevalence and incidence of type 1 diabetes in the world: a systematic review and meta-analysis.. Health Promot. Perspect..

[17] Lawrence J.M., Divers J., Isom S., Saydah S., Imperatore G., Pihoker C., Marcovina S.M., Mayer-Davis E.J., Hamman R.F., Dolan L., Dabelea D., Pettitt D.J., Liese A.D. (2021). Trends in prevalence of type 1 and type 2 diabetes in children and adolescents in the US, 2001-2017.. JAMA.

[18] Patterson C.C., Harjutsalo V., Rosenbauer J., Neu A., Cinek O., Skrivarhaug T., Rami-Merhar B., Soltesz G., Svensson J., Parslow R.C., Castell C., Schoenle E.J., Bingley P.J., Dahlquist G., Jarosz-Chobot P.K., Marčiulionytė D., Roche E.F., Rothe U., Bratina N., Ionescu-Tirgoviste C., Weets I., Kocova M., Cherubini V., Rojnic Putarek N., deBeaufort C.E., Samardzic M., Green A. (2019). Trends and cyclical variation in the incidence of childhood type 1 diabetes in 26 European centres in the 25 year period 1989–2013: a multicentre prospective registration study.. Diabetologia.

[19] Patterson C.C., Dahlquist G.G., Gyürüs E., Green A., Soltész G., EURODIAB Study Group (2009). Incidence trends for childhood type 1 diabetes in Europe during 1989–2003 and predicted new cases 2005–20: a multicentre prospective registration study.. Lancet.

[20] Moravej H., Rassafian M., Amirhakimi A., Ilkhanipour H., Yazdani N. (2020). A Survey on the Frequency of Type 1 Diabetes Mellitus and its Related Factors from 2004-2014.. J. Adv. Biomedi. Sci..

[21] Zamanfar D., Yazdani P., Aarabi M., Pournorooz H. (2018). The prevalence of type 1 diabetes in children of Mazandaran province.. Iran J. Health Sci..

[22] Pishdad G.R. (2005). Low incidence of type 1 diabetes in Iran.. Diabetes Care.

[23] Triolo T.M., Fouts A., Pyle L., Yu L., Gottlieb P.A., Steck A.K. (2019). Identical and nonidentical twins: risk and factors involved in development of islet autoimmunity and type 1 diabetes.. Diabetes Care.

[24] Hermann R., Knip M., Veijola R., Simell O., Laine A.P., Åkerblom H.K., Groop P.H., Forsblom C., Pettersson-Fernholm K., Ilonen J., FinnDiane Study Group (2003). Temporal changes in the frequencies of HLA genotypes in patients with Type 1 diabetes—indication of an increased environmental pressure?. Diabetologia.

[25] Fourlanos S., Varney M.D., Tait B.D., Morahan G., Honeyman M.C., Colman P.G., Harrison L.C. (2008). The rising incidence of type 1 diabetes is accounted for by cases with lower-risk human leukocyte antigen genotypes.. Diabetes Care.

[26] Rewers M., Ludvigsson J. (2016). Environmental risk factors for type 1 diabetes.. Lancet.

[27] Patelarou E., Girvalaki C., Brokalaki H., Patelarou A., Androulaki Z., Vardavas C. (2012). Current evidence on the associations of breastfeeding, infant formula, and cow’s milk introduction with type 1 diabetes mellitus: a systematic review.. Nutr. Rev..

[28] Rabiei S, Reza Rastmanesh (2012). The association of type 1 diabetes mellitus with nutrition style through the first 2 years of life in Iranian children.. J. US. China Med. Sci..

[29] Tanoey J., Gulati A., Patterson C., Becher H. (2019). Risk of type 1 diabetes in the offspring born through elective or non-elective caesarean section in comparison to vaginal delivery: A meta-analysis of observational studies.. Curr. Diab. Rep..

[30] Bodin J., Stene L.C., Nygaard U.C. (2015). Can exposure to environmental chemicals increase the risk of diabetes type 1 development?. BioMed Res. Int..

[31] Vakili R., Farivar K. (2002). Month of Birth in children and adolescents with type 1 diabetes mellitus in Mashhad.. Majallah-i Ghudad-i Darun/Riz va Mitabulism-i Iran.

[32] Al-Ghamdi A.H., Fureeh A.A. (2018). Prevalence and clinical presentation at the onset of type 1 diabetes mellitus among children and adolescents in AL-Baha region, Saudi Arabia.. J. Pediatr. Endocrinol. Metab..

[33] Çiçekli İ., Durusoy R. (2022). Breastfeeding, nutrition and type 1 diabetes: a case-control study in Izmir, Turkey.. Int. Breastfeed. J..

[34] Demir F., Günöz H., Saka N., Darendeliler F., Bundak R., Baş F., Neyzi O. (2015). Epidemiologic features of type 1 diabetic patients between 0 and 18 years of age in İstanbul city.. J. Clin. Res. Pediatr. Endocrinol..

[35] Lah Tomulić K., Matko L., Verbić A., Milardović A., Severinski S., Kolić I., Baraba Dekanić K., Šerifi S., Butorac Ahel I. (2022). Epidemiologic characteristics of children with diabetic ketoacidosis treated in a pediatric intensive care unit in a 10-year-period: single centre experience in Croatia.. Medicina (Kaunas).

[36] Szypowska A., Dżygało K., Wysocka-Mincewicz M., Mazur A., Lisowicz L., Ben-Skowronek I., Sieniawska J., Klonowska B., Charemska D., Nawrotek J., Jałowiec I., Bossowski A., Jamiołkowska M., Pyrżak B., Rogozińska I., Szalecki M. (2017). High incidence of diabetic ketoacidosis at diagnosis of type 1 diabetes among Polish children aged 10-12 and under 5 years of age: A multicenter study.. Pediatr. Diabetes.

[37] Magnus M.C., Olsen S.F., Granström C., Joner G., Skrivarhaug T., Svensson J. (2015). Infant growth and risk of childhood-onset type 1 diabetes in children from 2 Scandinavian birth cohorts.. JAMA pediatrics..

[38] Waernbaum I., Dahlquist G., Lind T. (2019). Perinatal risk factors for type 1 diabetes revisited: a population-based register study.. Diabetologia.

[39] Magnus M.C., Olsen S.F., Granstrom C., Lund-Blix N.A., Svensson J., Johannesen J., Fraser A., Skrivarhaug T., Joner G., Njølstad P.R., Størdal K., Stene L.C. (2018). Paternal and maternal obesity but not gestational weight gain is associated with type 1 diabetes.. Int. J. Epidemiol..

[40] Begum M., Pilkington R., Chittleborough C., Lynch J., Penno M., Smithers L. (2019). Caesarean section and risk of type 1 diabetes: whole-of-population study.. Diabet. Med..

[41] Khashan A.S., Kenny L.C., Lundholm C., Kearney P.M., Gong T., Almqvist C. (2014). Mode of obstetrical delivery and type 1 diabetes: a sibling design study.. Pediatrics.

[42] Dong J.Y., Zhang W., Chen J., Zhang Z.L., Han S.F., Qin L.Q. (2013). Vitamin D intake and risk of type 1 diabetes: a meta-analysis of observational studies.. Nutrients.

[43] Chia J., McRae J., Kukuljan S., Woodford K., Elliott R., Swinburn B. (2017). A1 beta-casein milk protein and other environmental pre-disposing factors for type 1 diabetes.. Nutrit. Diabetes..

[44] Siadat Z.D., Golshiri P., Hashemipour M., Ghaffarpour M.S., Ghaffarpour A.H. (2017). Assessment of risk factors for type I Diabetes Mellitus: A case-control study.. Isfahan Med. School J..

[45] Algert C.S., McElduff A., Morris J.M., Roberts C.L. (2009). Perinatal risk factors for early onset of Type 1 diabetes in a 2000–2005 birth cohort.. Diabet. Med..

[46] Głowińska-Olszewska B., Szabłowski M., Panas P., Żoła̧dek K., Jamiołkowska-Sztabkowska M., Milewska A.J., Kadłubiska A., Polkowska A., Łuczyński W., Bossowski A. (2020). Increasing co-occurrence of additional autoimmune disorders at diabetes type 1 onset among children and adolescents diagnosed in years 2010–2018—single-center study.. Front. Endocrinol. (Lausanne).

[47] Burbaud M., Renard E., Jellimann S., Luc A., Di Patrizio M., Remen T., Legagneur C. (2022). Additional autoimmune diseases associated with type 1 diabetes in children and adolescents: A French single-center study from 2014 to 2021.. Arch. Pediatr..

